# Insecticide resistance and malaria transmission indicators in *Anopheles gambiae* s.l. in Bobo-Dioulasso, Burkina Faso: implications for vector control strategies

**DOI:** 10.1186/s41182-025-00853-y

**Published:** 2025-11-19

**Authors:** Miriam Félicité Amara, Moussa Namountougou, Hamadou Konaté, Kouamé Wilfred Ulrich Kouadio, Koudraogo Bienvenue Yaméogo, Sadapawindé Thérèse Kagoné, Abdoulaye Diabaté, Olivier Gnankine

**Affiliations:** 1 Unité de Formation Et de Recherche en Sciences de La Vie Et de La Terre, Université Nazi BONI, Bobo-Dioulasso, Burkina Faso; 2 Centre MURAZ, Bobo-Dioulasso, Burkina Faso; 3 Institut de Recherche, en Sciences de La Santé-Direction Régionale Ouest (IRSS-DRO), Bobo-Dioulasso, Burkina Faso; 4 Unité de Formation Et de Recherche en Sciences de La Vie Et de La Terre, Université Joseph KI-ZERBO, Ouagadougou, Burkina Faso

**Keywords:** Malaria, *Anopheles gambiae* s.l., Insecticide, Synergist, Resistance, SIR, EIR, Burkina Faso

## Abstract

**Background:**

In the context of intensified malaria control efforts in Burkina Faso, this study assessed i) the insecticide resistance status of *Anopheles gambiae* sensu lato and ii) key entomological indicators of malaria transmission in Bobo-Dioulasso.

**Methods:**

World Health Organization–standard susceptibility bioassays were conducted on *Anopheles* populations collected from six neighborhoods (Kua, Sarfalao, Sabaribougou, Dogona, Farakan and Kodeni). The bioassays tested six insecticides organochlorines (4%dichlorodiphenyltrichloroethane), organophosphates (1.25% pirimiphos-methyl), pyrethroids (0.75%permethrin, 0.05% deltamethrin, 0.05% alpha-cypermethrin), and carbamates (0.1% bendiocarb). Synergist bioassays using piperonyl butoxide (PBO) were also performed to investigate metabolic resistance mechanisms, and *Plasmodium* infection rates were determined via Polymerase Chain Reaction.

**Results:**

Overall, data revealed high resistance levels to dichlorodiphenyltrichloroethane and pyrethroids, which are associated with moderate or higher frequencies of knockdown resistance mutations (L995F and L995S). Fortunately, a susceptibility to bendiocarb and pirimiphos-methyl was found in the majority of localities. The restoration of pyrethroid susceptibility following piperonyl butoxide pre-exposure suggests the involvement of metabolic resistance mechanisms. Analysis of 622 specimens from the *Anopheles gambiae* complex revealed a predominance of *An. arabiensis* (90.8%), followed by *An. gambiae* s.s. and *An. coluzzii*. Sporozoite infection rates varied by species, reaching 45% in *An. coluzzii*, 27.4% in *An. arabiensis*, and 16.2% in *An. gambiae* s.s*.* The overall entomological inoculation rate (EIR) was estimated at 10.6 infectious bites per person during the study period. *Anopheles arabiensis* contributed most of these bites (91.2%), highlighting its central role in malaria transmission in Bobo-Dioulasso.

**Conclusions:**

Despite insecticide resistance, *Anopheles* populations exhibited high *Plasmodium* infection rates, indicating ongoing transmission. These findings emphasize the urgent need for sustained entomological surveillance and resistance management to guide and optimize insecticide-based malaria control strategies.

## Background

Malaria remains an endemic disease worldwide, with control strategies primarily based on the use of long-lasting insecticidal nets (LLINs) and indoor residual spraying (IRS) [1, 2]. Four major classes of insecticides are used in vector control programs: pyrethroids, organophosphates, carbamates, and organochlorines [3, 4]. Pyrethroids are currently the only class recommended by the World Health Organization (WHO) for insecticide treatment net (ITN), due to rapid action, and low toxicity to humans [5]. However, widespread resistance to all four classes of insecticide has been reported in *Anopheles gambiae* s.l. across sub-Saharan Africa. This has seriously undermined the effectiveness of interventions based on long-lasting insecticide-treated nets (LLINs) and indoor residual spraying (IRS) [6–8]. These tools, which have been instrumental in significantly reducing *Plasmodium falciparum* infection prevalence over the past decade, but they are now facing growing limitations in their efficacy [9, 10]. In response to this threat, one of the most promising approaches is to combine two active ingredients with different modes of action in a single LLIN. This combined strategy could enhance the sustainability of interventions and improve the management of insecticide resistance in malaria vectors [11, 12]. The development of insecticide resistance among malaria vectors is a dynamic and unstable phenomenon. Its intensity that can rapidly increase under multiple selection pressures particularly those induced by public health interventions and intensive agricultural practices that use insecticides belonging to the same chemical classes [13–15]. Several authors have shown the role of agricultural practices in selecting *An. gambiae* resistant populations to chemicals. According to Amara et *al*., a study conducted in the city of Bobo-Dioulasso revealed that the *An. gambiae* s.l. complex is primarily dominated by *An. arabiensis*, which accounted for the majority of mosquitoes collected across various urban districts [16]. This predominance suggests a specific ecological adaptation of *An. arabiensis* to local urban conditions, which has important implications for targeted vector control strategies [15, 16]. However, a previous study conducted in Dioulassoba, a district of Bobo-Dioulasso, Burkina Faso, revealed significant pyrethroid resistance among *An. Arabiensis* populations. Of the *An. gambiae* s.l. complex mosquitoes collected, over 31% were identified as *An. arabiensis* (Namountougou et *al.* [15]). These populations exhibited mortality rates below 30% following exposure to deltamethrin, indicating high phenotypic resistance. Furthermore, the *kdr* 995F mutation, which is known to confer resistance to pyrethroids, was detected at a high frequency (0.98) [6]. The city located in the Sudanian zone of Burkina Faso, exhibits highly heterogeneous malaria transmission patterns. Previous studies have shown that transmission is seasonal, peaking during the second half of the rainy season, and varies significantly across urban districts [17]. While central urban areas report very low transmission levels (as low as 0.14 infectious bites per person per year), peripheral neighborhoods such as Colma-Nord can reach much higher rates, up to 4.6 infectious bites per person annually. This variability is closely linked to the availability of larval habitats and local ecological conditions [18, 19]. From a parasitological perspective, *Plasmodium falciparum* remains the predominant species responsible for malaria transmission in the region, consistent with findings across West Africa. Other *Plasmodium* species, such as *P. malariae* and *P. ovale*, are rarely detected and play only a marginal role in local transmission dynamics [16].

This study aims to assess the status of insecticide resistance in *Anopheles gambiae *sensu lato populations in Bobo-Dioulasso, Burkina Faso, and to evaluate key entomological indicators of malaria transmission across different urban and peri-urban settings. By combining resistance profiling with transmission metrics, such as vector density, sporozoite rates, and biting behavior, the study seeks to generate evidence that informs and optimizes local vector control strategies. Understanding the spatial heterogeneity of transmission and resistance patterns is essential for tailoring interventions to the ecological realities of each neighborhood.

## Methods

### Study area

**Fig. 1:**
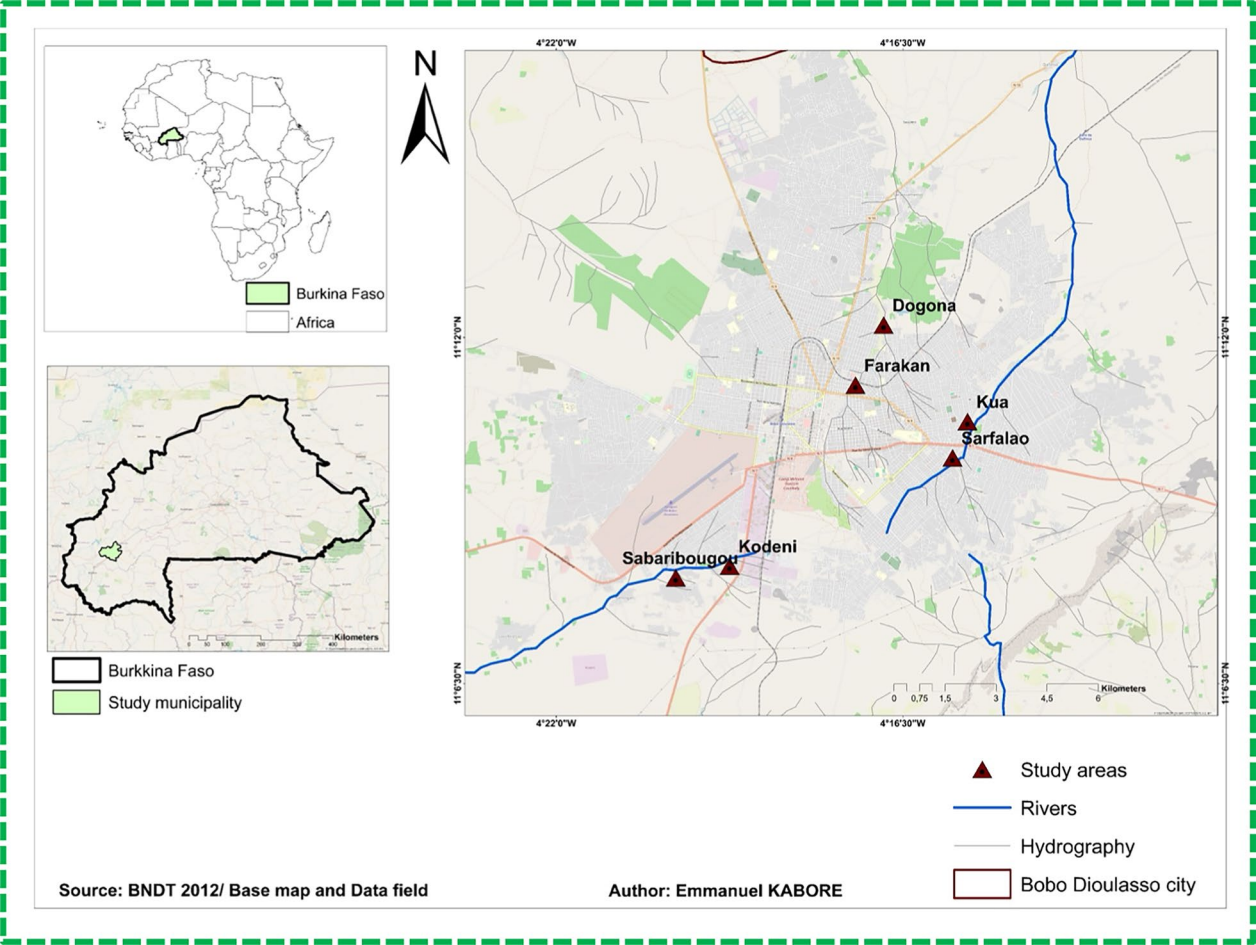
Study areas: Bobo-Dioulasso (western Burkina Faso)

The study was conducted in six districts of Bobo-Dioulasso, located in the Hauts-Bassins region in the southwestern part of Burkina Faso (11°10′37″ N, 4°17′52″ W) (Fig. 1). The city of Bobo Dioulasso is the second-largest urban area in the country, which is characterized by a rainy season extending from May to September, and an average annual rainfall exceeding 1,200 mm. The city experiences a southern Sudanian climate and is marked by intensive agricultural activity in peri-urban areas. The selected districts are intersected by permanent watercourses, around which vegetable farming zones (notably in Kua, Sarfalao, and Sabaribougou) and rice cultivation areas (in Dogona, Farakan, and Kodeni) have developed. Site selection was based on a combination of hydrological, agricultural, and entomological criteria, to capture the diversity of transmission contexts. These agricultural developments, combined with the proximity of human dwellings to humid environments, promote the formation of numerous larval habitats for malaria vector mosquitoes.

### Collection and rearing mosquito larvae

During the 2025 rainy season from May to July, *Anopheles* larvae were collected from natural breeding sites using the standard dipping method**.** At each district, sampling was conducted across multiple distinct larval habitats, including all developmental stages. Larvae samples were then pooled by locality. The larvae were reared at the IRSS/Centre Muraz insectary in Bobo-Dioulasso under controlled environmental conditions (temperature: 27–30 ± 1 °C; relative humidity: 80 ± 10%; photoperiod: 12 h of light and 12 h of darkness, including a 1-h transition at dawn and dusk), until adult emergence (F0). They were fed daily with Tetramin® fish food. After emergence, adult female mosquitoes were maintained on a 10% sugar solution in preparation for insecticide susceptibility testing. Morphological identification of the specimens was then performed using the taxonomic keys of Gillies and De Meillon, [20].

### Insecticide susceptibility tests

Insecticide susceptibility tests were conducted on 2–5-day-old, non-blood-fed adult female mosquitoes of the *Anopheles gambiae* complex using the WHO tube test protocol [21]. The test papers were impregnated with pyrethroids (alpha-cypermethrin 0.05%, deltamethrin 0.05%, and permethrin 0.75%), an organochlorine (DDT 4%), a carbamate (bendiocarb 0.1%), and an organophosphate (pirimiphos-methyl 0.25%). The *An. gambiae* s.s. Kisumu strain ﻿has been tested on all insecticides impregnated papers as a control. Four replicates, each containing 20–25 mosquitoes, were introduced into WHO test tubes and observed for 60 min to ensure post-transfer recovery and to exclude any handling-related mortality. In addition, all field mosquitoes and the Kisumu strain were exposed for 1 h to non-impregnated papers. Environmental conditions were standardized at a temperature of 25 ± 2 °C and relative humidity between 70 and 80%, to ensure optimal testing conditions. Mosquitoes were then exposed to insecticides for a strictly controlled duration of 60 min. Afterward, they were transferred to observation tubes and kept for 24 h under the same thermo-hygrometric conditions, with access to a 10% sugar solution provided on cotton pads. Mortality was assessed 24-h post-exposure. Alive and dead mosquitoes were counted separately and preserved in Eppendorf tubes containing silica gel covered with cotton, according to sampling site and insecticide type. The samples were stored at − 20 °C for molecular analyses, including subspecies identification and detection of resistance-associated mutations (*West* and *East kdr*).

### Synergist-based bioassays

To investigate the potential role of detoxification enzymes in pyrethroid resistance, complementary bioassays were conducted using the synergist Piperonyl Butoxide (PBO) at 4%, known to inhibit monooxygenases and, to a lesser extent, esterases [22]. Non-blood-fed *Anopheles gambiae* s.l. females aged 2–5 days were pre-exposed to PBO for 1 h before being transferred onto insecticide-impregnated papers (alpha-cypermethrin 0.05%, deltamethrin 0.05%, permethrin 0.75%). Control mosquitoes underwent an identical pre-exposure on untreated papers. After being exposed, mosquitoes were placed in observation tubes containing cotton soaked in a 10% sugar solution and maintained under controlled conditions for 24 h prior to mortality assessment. The same procedures were applied to the susceptible *An. gambiae* s.s. Kisumu strain. Each treatment was replicated four times, with batches of 20 to 25 mosquitoes per replicate.

### Human landing catch (HLC)

A cross-sectional entomological survey was conducted between July and September 2024 in the same areas targeted for the bioassays. Human landing catches were performed in accordance with the WHO standard protocol [21]. Informed consent was obtained from household heads, in consultation with local authorities, including traditional leaders and designated focal points for each study site. In each of the six selected neighborhoods, eight trained adult volunteers were recruited as mosquito collectors. Two collectors (one indoors and one outdoors) were assigned to each of the four sampled households per neighborhood. Collection teams operated in two shifts: the first from 6:00 p.m. to 1:00 a.m., and the second from 1:00 a.m. to 9:00 a.m. Collectors sat on chairs with their legs exposed and used flashlights to visually detect and capture mosquitoes landing on their limbs before blood-feeding, using hemolysis tubes.

To minimize potential bias related to individual attractiveness to mosquitoes, collectors alternated between indoor and outdoor positions every hour. Each collection site was supervised by a technician from the Institut de Recherche en Sciences de la Santé (IRSS)/Centre MURAZ, assisted by a local field agent. Mosquitoes collected in hemolysis tubes were grouped by hour of collection into separate bags and transported daily to the IRSS/Centre MURAZ entomology laboratory. Upon arrival at the laboratory, specimens were immediately sorted. Each mosquito was individually transferred into a labeled Eppendorf tube containing silica gel to ensure proper desiccation and preservation for subsequent molecular analyses. Morphological identification of mosquito species was performed using a binocular magnifying glass (AmScope; United Scope LLC) and based on the taxonomic keys of Gillies et De Meillon (1968).

### Molecular processing

#### Anophelines’ molecular characterization

Genomic DNA was extracted from homogenized mosquitoes using the cetyltrimethylammonium bromide (CTAB) method (2% CTAB buffer), adapted from the works of Cornel et al. [23]. The extraction process included chloroform purification followed by isopropanol precipitation. Then, identification of sibling species within the *Anopheles gambiae* complex was performed using allele-specific polymerase chain reactions (PCR), targeting insertion polymorphisms of the SINE200 retro transposable element, as described by Santolamazza et *al.*, (2008) [24]. The primers used were: S200X 6.1F: TCG–CCT–TAG–ACC–TTG–CGT–TA; S200X 6.1R: CGC–TTC–AAG–AAT–TCG–AGA–TAC. PCR reactions were carried out in 20 µL volumes under standard thermal cycling conditions. Amplified products were separated on 2% agarose gels to distinguish target species, with expected band sizes of 479 base pair (bp) for *An. coluzzii*, 249 bp for *An. gambiae*, and 223 bp for *An. arabiensis*.

### Detection of *kdr* mutation (Kdr-West and Kdr-East)

The kdr L995F mutation was detected using an allele-specific PCR (AS-PCR) protocol described by Martinez-Torres et *al*., [25]. The primers Agd1 (5′-ATA GAT TCC CCG ACC ATG-3′) and Agd2 (5′-AGA CAA GGA TGA TGA ACC-3′) were used to amplify a common fragment of 293 base pairs (bp) in mosquitoes belonging to the *Anopheles gambiae s.l.* complex. To distinguish between alleles, the specific primers Agd3 (5′-AAT TTG CAT TAC TTA CGA CA-3′) and Agd4 (5′-CTG TAG TGA TAG GAA ATT TA-3′) were used to identify the L995F mutation. In parallel, detection of the kdr L995S mutation, characteristic of East Africa, was performed using the primers Agd1, Agd2, Agd4, and Agd5 (5′-TTT GCA TTA CTT ACG ACT G-3′), following the protocol adapted from Ranson et *al*., (2000) and validated by Verhaeghen et *al*., [26, 27]. This combination specifically targets the substitution of leucine by serine at codon 995 of the voltage-gated sodium channel gene. The primers were used to amplify two distinct fragments: a 195 bp product corresponding to the mutated (resistant) allele, and a 137 bp product corresponding to the non-mutated (susceptible) allele. PCR products were separated on 2% agarose gels, enabling clear visualization of the banding patterns and accurate genotype determination for each individual.

Three genotypes were considered in the interpretation of kdr L995F and L995S mutations: homozygous resistant (RR): bands at 293 bp and 195 bp, heterozygous (RS): bands at 293 bp, 195 bp, and 137 bp and homozygous susceptible (SS): bands at 293 bp and 137 bp. The presence of the common 293 bp fragment, amplified by primers Agd1 and Agd2, was essential to validate each PCR reaction and confirm the integrity of the amplification process.

### Detection of *Plasmodium* infections

Detection of *Plasmodium* species was performed using the head and thorax of gravid and semi-gravid female *Anopheles* mosquitoes collected through human landing catches (HLC), following the PCR protocol described by Boonma and collaborators [28]. PCR amplification was carried out in a 25 µL reaction volume. The thermal cycling conditions included an initial denaturation at 95 °C for 5 min, followed by 35 amplification cycles: denaturation at 95 °C for 30 s, annealing at 58 °C for 45 s, and elongation at 72 °C for 1 min. A final extension step was performed at 72 °C for 5 min. PCR products were separated by electrophoresis on a 2% agarose gel stained with ethidium bromide and visualized under UV illumination. Fragment sizes were determined by comparison with a 100 bp molecular weight marker. The expected amplicon sizes for each *Plasmodium* species were 276 bp, 376 bp and 411 bp for *P. falciparum, P. ovale* and *P. malariae,* respectively.

### Statistical analyses

Mortality rates and 95% confidence intervals for the WHO susceptibility tests were calculated using the exact binomial method with RStudio software (version 4.4.2). The frequency of L995F and L995S mutations was calculated using the following formula: F(kdr) or f(995F) or f(995L) = (2nRR + nRS)/2N, where *nRR* represents the number of homozygous resistant individuals, *nRS* the number of heterozygotes, and *N* the total number of specimens analyzed. The criteria for assessing the resistance status of a mosquito population to insecticide are as follows [3].

• Mortality rate between 98 and 100%: susceptible mosquito population.
• Mortality rate between 90 and 97%: mosquito population with possible resistance.
• Mortality rate below 90%: resistant mosquito population.

Pearson correlation tests were performed to compare the abundance of mosquito species collected indoors and outdoors. A significance threshold of α = 0.05 was adopted, and results with *p* values below 0.05 were considered statistically significant.

Then, the following entomological parameters were calculated:

- Anopheline density (ma):

ma = Total number of *Anopheles* mosquitoes captured/(Number of collectors x Number of capture days).

(m = anopheline density; a = The daily probability that a vector bites the host, expressed as the ratio of the anthropophily rate to the duration of the gonotrophic cycle (in days).

- Sporozoite infection rate (SIR):

SIR = (Number of anopheles infected with *P. falciparum*/Total number of anopheles analyzed) × 100.

- Entomological inoculation rate (EIR):

EIR = ma x SIR;

## Results

### Distribution of *Anopheles* complex species

**Fig. 2:**
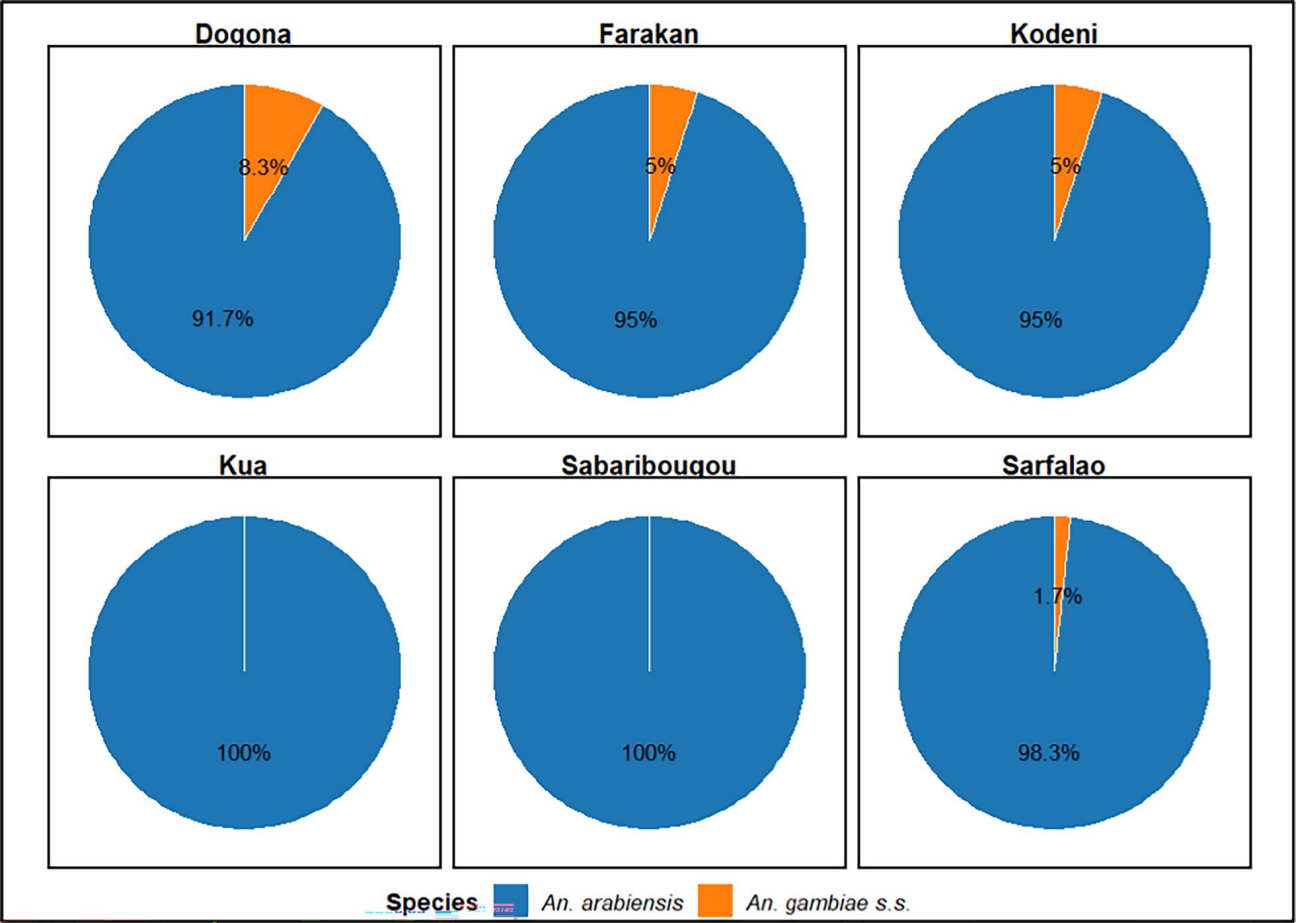
Distribution of *Anopheles gambiae* s.l species in the study area

A total of 360 *An. gambiae* s.l individuals from bioassays were analyzed by PCR to identify *Anopheles gambiae* complex species. Of these, *An. arabiensis* (96.67%, 348/360) and *An. gambiae* s.s. (3.33%, 12/360) were identified as members of the *An. gambiae* complex. Our data shown a clear predominance of *An. arabiensis* across all surveyed localities, with proportions ranging from 91.7% in Dogona to 100% in Kua and Sabaribougou. *An. gambiae* s.s. was present, at low frequencies, below 10%, in most sites and was completely absent in Kua and Sabaribougou (Fig. 2).

### Susceptibility status of *Anopheles gambiae* to PBO + pyrethroids

Exposure of *Anopheles gambiae* to insecticide combinations enhanced with the synergist PBO (Piperonyl butoxide) resulted in a notable increase in mortality rates across all surveyed localities. The combinations PBO + permethrin, PBO + deltamethrin, and PBO + alpha-cypermethrin proved more effective than the insecticides used alone, indicating partial inhibition of metabolic resistance mechanisms in most sites. PBO restores either partially or totally the susceptibility to pyrethroids according to investigated localities.

**Fig. 3:**
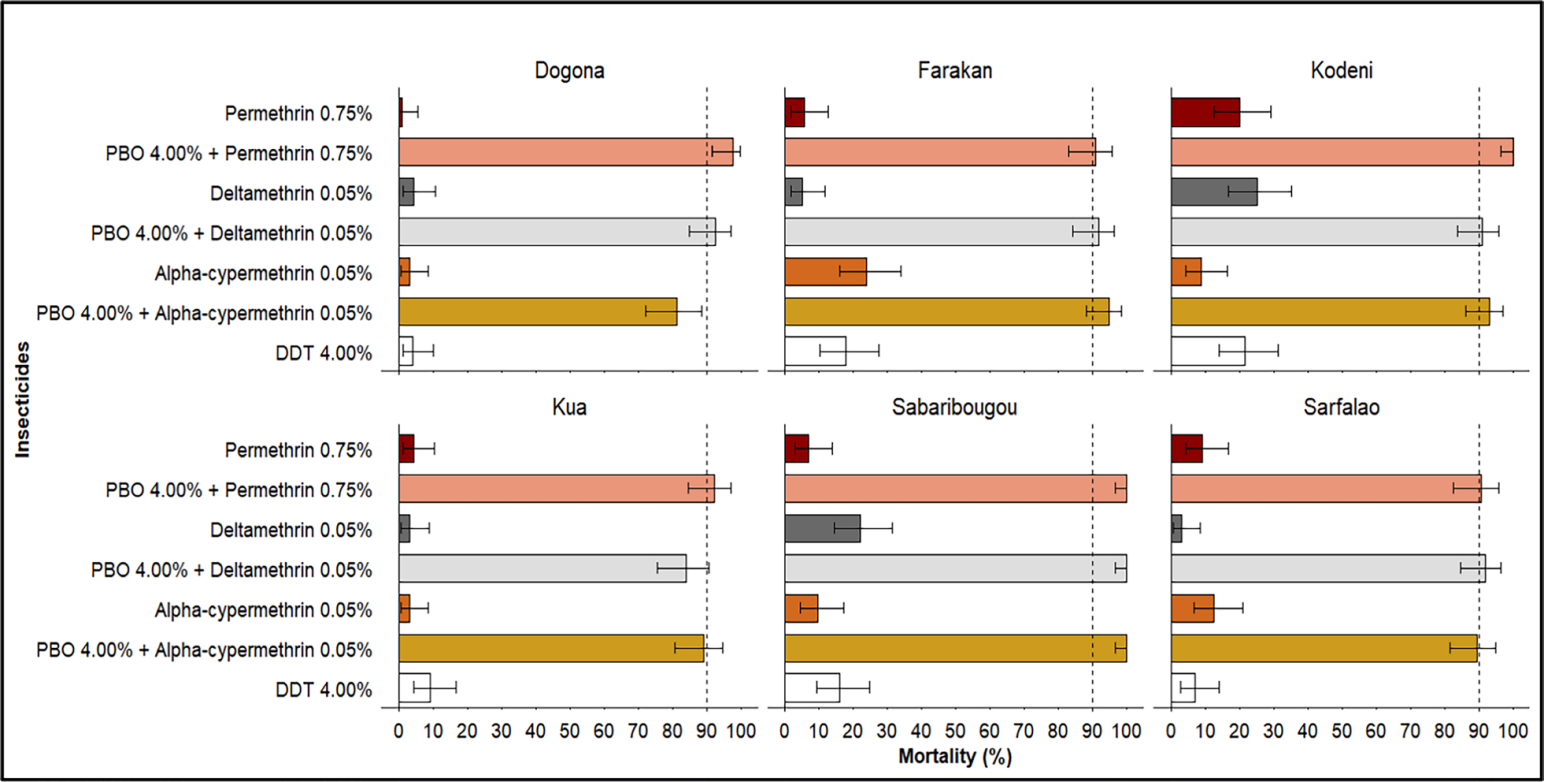
Mortality rates of *Anopheles gambiae* s.l. following exposure to pyrethroid and pyrethroid–PBO combinations

However, in Sabaribougou, the markedly improved efficacy observed with PBO-containing formulations suggests a predominant, if not exclusive, involvement of metabolic resistance mechanisms in the local mosquito population (Fig. 3).

### Susceptibility status of species to organophosphates and carbamates

**Fig. 4:**
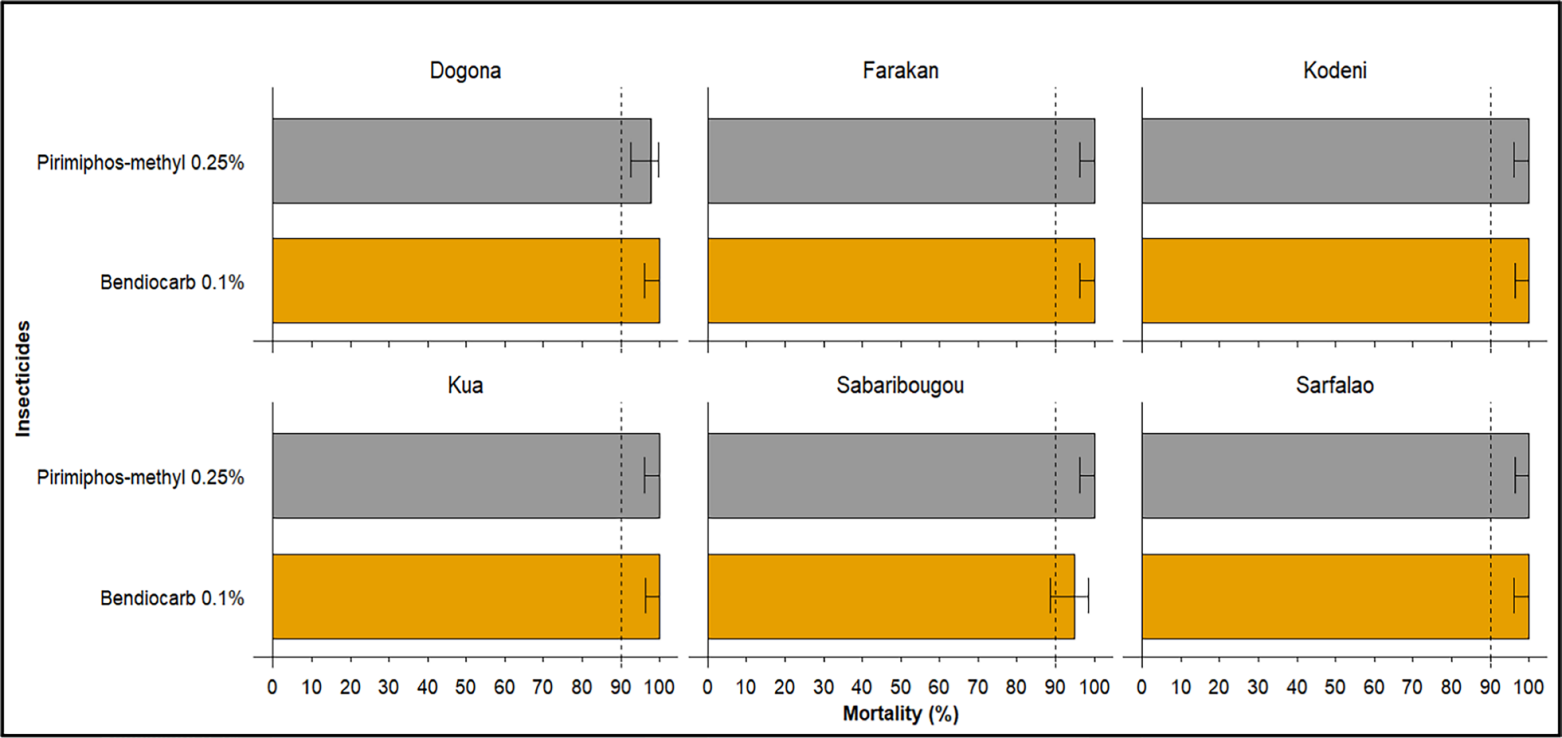
Mortality rates of *Anopheles gambiae* s.l. after exposition to organophosphates and carbamates in the study sites

The tests demonstrated consistently high efficacy of pirimiphos-methyl and bendiocarb at most of the surveyed sites. The mortality rates exceeded the WHO’s recommended threshold of 98% to confirm susceptibility. This observation suggests no significant resistance to these two insecticides within local *An. gambiae* s.l. populations (Fig. 4).

### Distribution of *Kdr-West* and *Kdr-East* mutation frequencies

The *Kdr-West* (L995F) mutation was prevalent across all study sites, in *An. arabiensis*, with frequencies varying according to both species and areas. The frequencies ranged from 0.533 in Sabaribougou to 0.745 in Sarfalao, indicating strong selection pressure likely driven by pyrethroid use. The *Kdr-East L995S* mutation was also detected though less frequently (0.118 in Sarfalao and 0.421 in Kodeni). This suggests the co-circulation of both resistance alleles, with a clear predominance of the L995F mutation. *Kdr-East* frequencies were more variable (ranging from 0 in Sarfalao to 0.666 in Kodeni) but remained lower than those of L995F (Table 1).

**Table 1:** Allelic frequencies of *Kdr-West* and *Kdr-East* in *Anopheles gambiae* s.l. populations of Bobo-Dioulasso

Sites	Species		Genotypes *Kdr-West*					Genotypes *Kdr-East*				
		N	995L 995L	995L 995F	995F 995F	f(995F)	p(HW)	995L 995L	995L 995S	995S 995S	f(995S)	p(HW)
Dogona	*An. arabiensis*	55	13	20	22	0.581	0.9395	31	17	7	0.281	0.9131
	*An. gambiae* s.s	5	0	2	3	0.8	0.9905	4	0	1	0.1	0.9747
Farakan	*An. arabiensis*	57	15	14	28	0.614	0.9998	41	9	7	0.01	0.9996
	*An. gambiae* s.s	3	0	1	2	0.833	0.3464	0	3	0	0.5	0.9586
Kodeni	*An. arabiensis*	57	7	18	32	0.719	0.8922	21	24	12	0.421	0.6965
	*An. gambiae* s.s	3	0	3	0	0.5	0.9586	0	2	1	0.666	0.6937
Kua	*An. arabiensis*	60	10	20	30	0.666	0.9446	37	20	3	0.216	0.1103
Sabaribougou	*An. arabiensis*	60	20	16	24	0.533	0.9998	37	19	4	0.225	0.514
Sarfalao	*An. arabiensis*	59	8	14	37	0.745	0.9942	45	14	0	0.118	0.8308
	*An. gambiae* s.s	1	0	0	1	1	< 0.0001	1	0	0	0	< 0.0001

N: number of mosquitoes; 995L 995L: sensitive homozygote; 995F 995L or 995L et 995S: heterozygote; 995F 995F or 995S 995S: resistant homozygote; f(995F): frequency of the kdr-west mutation; f(995S): frequency of the kdr-east mutation; p(HW): Hardy–Weinberg test for rejection or acceptance of the Hardy–Weinberg equilibrium hypothesis

### Abundance of *Anopheles* mosquitoes collected both indoor vs outdoor by HLC sporozoite

A total of 622 mosquitoes from HLC were identified, including 283 collected indoors and 339 outdoors. Significative difference was found between the collected mosquitoes indoor and outdoor (*p* value 0.016 cal). Among the specimens captured indoors, *An. arabiensis* was predominant, accounting for 88% (n = 249), followed by *An. gambiae* s.s. at 7.8% (n = 22) and *An. coluzzii* at 4.2% (n = 12). In the outdoor environment, *An. arabiensis* remained the most prevalent species, with a slightly higher proportion of 93.2% (n = 316), while *An. gambiae* s.s. and *An. coluzzii* were less frequent, representing 4.4% (n = 15) and 2% (n = 8), respectively (Table 2).

**Table 2:** Distribution of *Anopheles gambiae* s.l. collected indoors and outdoors

Sites	Indoor n (%)			Total	Outdoor n (%)			Total
	*An. arabiensis*	*An. gambiae* s.s	*An. coluzzii*		*An. arabiensis*	*An. gambiae* s.s	*An. coluzzii*	
Sarfalao	54 (84.376)	7 (10.937)	3 (4.687)	64 (22.615)	46 (97.872)	1 (2.128)	0 (0.000)	47 (13.864)
Kua	26 (89.655)	1 (3.448)	2 (6.897)	29 (10.247)	75 (89.286)	5 (5.952)	4 (4.762)	84 (24.779)
Sabaribougou	54 (93.104)	2 (3.448)	2 (3.448)	58 (20.494)	34 (94.444)	1 (2.778)	1 (2.778)	36 (10.619)
Kodeni	20 (74.074)	5 (18.519)	2 (7.407)	27 (9.541)	73 (92.405)	6 (7.595)	0 (0.000)	79 (23.304)
Farakan	27 (90.000)	3 (10.000)	0 (0.000)	30 (10.601)	25 (96.154)	0 (0.000)	1 (3.846)	26 (7.670)
Dogona	68 (90.667)	4 (5.333)	3 (4.000)	75 (26.502)	63 (94.030)	2 (2.985)	2 (2.985)	67 (19.764)
Total	249 (88)	22 (7.7)	12 (4.2)	283 (100)	316 (93.2)	15 (4.4)	8 (2.4)	339 (100)

### Sporozoite infection rate (SIR) and entomological inoculation rate (EIR)

A total of 622 mosquitoes from the *Anopheles gambiae* complex were analyzed for sporozoite detection. The observed sporozoite infection rates (SIR) were 27.4% for *An. arabiensis*, 45% for *An. coluzzii*, and 16.2% for *An. gambiae* s.s. The overall entomological inoculation rate (EIR) was estimated at 10.6 infectious bites per person, with *An. arabiensis* exhibiting the high rate (EIR = 9.7). The contributions from *An. coluzzii* and *An. gambiae* s.s. were more modest, at 0.56 and 0.37, respectively. Marked spatial variability was observed. The district of Sabaribougou recorded the highest EIR (3.5) and was associated with high infection rates in *An. coluzzii* (100%) and *An. gambiae* s.s. (66.7%). The districts of Kua (EIR = 2.4) and Kodeni (EIR = 1.5) also showed notable transmission, mainly attributed to *An. arabiensis*. In contrast, Farakan (EIR = 0.6) and Dogona (EIR = 1.3) exhibited lower transmission levels, although *An. coluzzii* occasionally showed high infection rates in these areas (Table 3).

**Table 3:** Sporozoite infection rate (SIR) and entomological inoculation rate (EIR) recorded in the *Anopheles gambiae* complex

Sites	*An. arabiensis*			EIR	*An. coluzzii*			EIR	*An. gambiae* s.s			EIR	EIR (T)
	Tested	ma	SIR (%)		Tested	ma	SIR (%)		Tested	ma	SIR (%)		
Sarfalao	99	0.062	17.17	1.062	3	0.018	33.33	0.062	8	0.005	25.00	0.125	1.249
Kua	99	0.062	36.36	2.250	6	0.003	50.00	0.187	6	0.000	0.00	0.000	2.412
Kodeni	98	0.061	22.44	1.375	2	0.001	50.00	0.062	11	0.006	09.09	0.062	1.499
Sabaribougou	86	0.053	59.30	3.187	3	0.001	100	0.187	3	0.002	66.67	0.125	3.499
Farakan	52	0.032	17.31	0.562	1	0.0001	100	0.062	3	0.00	0.00	0.000	0.624
Dogona	131	0.082	15.27	1.250	5	0.000	0.00	0.000	6	0.004	16.67	0.062	1.312
Total	565	0.353	27.43	9.687	20	0.012	45.00	0.562	37	0.023	16.21	0.375	10.624

*ma* anopheline density, *SIR* sporozoite infection rates, *EIR* entomological inoculation rate;(T): total EIR

## Discussion

Understanding the current level of insecticide resistance in malaria vectors belonging to the *Anopheles gambiae* complex in Bobo-Dioulasso, Burkina Faso, is prerequisite for developing efficient vector control strategies. Thus, the data obtained from this study provides a foundation for further reflection on the current state of insecticide resistance in malaria vectors in this region. They also extend knowledge of local malaria transmission.

The predominance of *An. Arabiensis* associated with the high prevalence of *Kdr* mutations, the variability in mortality rates across insecticides and study sites, reflects a complex entomological dynamic shaped by local selective pressures. These updated data are essential for refining vector control strategies, particularly by considering species- and locality-specific resistance profiles. They also emphasize the importance of incorporating additional tools, such as PBO-based formulations or alternative insecticides, into malaria control programs to maintain intervention effectiveness and prevent the development of resistance. The predominance of *An. arabiensis* across study sites can be explained by its remarkable ecological plasticity and ability to adapt to semi-arid environments and temporary larval habitats, which are abundant during the rainy season. Unlike *An. gambiae s.s.*, *An. arabiensis* is more effective at tolerating high temperatures and low humidity levels, giving it a competitive advantage in dry climates [29].

*An. arabiensis* is known to exhibit relative zoophily and a pronounced exophilic tendency, which reduce its exposure to indoor interventions, such as residual spraying and insecticide-treated nets [30]. This behavioral shift has been documented in multiple settings, including Guinea–Bissau and southeastern Tanzania, where *An. arabiensis* dominated outdoor collections and contributed significantly to residual transmission [31, 32]. However, the findings of this study, which show no significant difference in the proportions of *An. arabiensis* collected indoors and outdoors, highlighting its ability to adapt its biting behavior. Furthermore, genetic studies have highlighted the strong resilience of *An. arabiensis* populations in the face of environmental pressures and vector control interventions [33]. Finally, the increasing resistance to insecticides, particularly pyrethroids, may contribute to the persistence and dominance of *An. arabiensis* in certain areas [34].

In contrast, *An. gambiae s.s.* and *An. coluzzii* were encountered more frequently indoors, albeit at lower overall proportions. These species are traditionally considered endophilic and anthropophilic, traits that make them more susceptible to indoor interventions. However, their reduced presence in this study may reflect ecological displacement or behavioral changes in response to sustained vector control efforts, as has been observed in other West African contexts. The observed species distribution has important implications for malaria control strategies. The occurrence of *An. arabiensis* outdoors highlights the necessity of complementing indoor interventions with approaches that target outdoor biting. These approaches include the use of spatial repellents and community-based environmental modifications, such as outdoor livestock rearing, which may serve as alternative hosts for vector mosquitoes in outdoor settings. Moreover, the persistence of *An. gambiae* s.s*.* and *An. coluzzii* indoors suggests that ITNs and IRS are still relevant, though they may need to be optimized to address changing vector behaviors [35].

Molecular resistance observed in *An. Arabiensis*, although generally less pronounced than in *An. gambiae* s.s., but remains a concern in several African regions, notably in Ethiopia, Sudan, and Zimbabwe, where moderate-to-high levels of resistance have been documented [36–38]. The observation of widespread resistance to pyrethroids particularly permethrin and deltamethrin in this study area confirms a worrying trend already well-documented across West and Central Africa. This resistance appears to be primarily driven by metabolic mechanisms and behavioral adaptations, such as exophily and zoophily that reduce exposure to insecticides used in domestic settings. These traits complicate both the detection and management of resistance, particularly in areas, where pyrethroids were intensively used through long-lasting insecticidal nets (LLINs) and indoor residual spraying (IRS) campaigns. Recent studies have confirmed that the efficacy of these insecticides is significantly reduced in regions with a high prevalence of resistance [34, 39]. To address this issue, it is crucial to implement alternative strategies, such as using dual-active nets that combine a pyrethroid with a synergist like piperonyl butoxide (PBO), or deploying insecticides with different modes of action that are not cross-resistant with conventional compounds [40]. Strengthening entomological and molecular surveillance also remains critical for tracking the evolution of resistance markers and adapting interventions in real time. Combination of pyrethroids with the synergist piperonyl butoxide (PBO), specifically PBO + permethrin, PBO + deltamethrin, and PBO + alpha-cypermethrin offer superior efficacy compared to pyrethroid-only formulations, as indicated by the increased mosquito mortality observed across most sites. PBO functions by blocking the enzymatic activity of detoxification systems, thereby allowing pyrethroids to retain their lethal effect on resistant mosquitoes [41].

At the Sabaribougou site, the markedly improved efficacy of PBO-based formulations suggests a predominant, if not exclusive, role of metabolic resistance mechanisms. This hypothesis is supported by the absence of high-frequency *kdr* mutations in local populations associated with elevated expression levels of metabolic resistance genes, as documented in similar West African contexts [42]. These observations highlight the importance of using dual-active long-lasting insecticidal nets (LLINs–PBO) in areas, where metabolic resistance is prevalent. They also reinforce the recommendations of the WHO Global Plan for Insecticide Resistance Management (GPIRM), which advocates for the integration of synergized formulations into vector control programs when metabolic resistance mechanisms are confirmed [22]. Finally, these results underscore the need to strengthen molecular and enzymatic surveillance to guide the selection of insecticides based on local resistance profiles.

Fortunately, tests conducted with organophosphates and carbamates revealed consistently high efficacy for pirimiphos-methyl and bendiocarb, with mortality rates exceeding the WHO’s recommended threshold of 98% to confirm vector susceptibility [22]. This susceptibility suggests that, despite the widespread resistance to pyrethroid, organophosphates and carbamates remain operationally effective in several entomological settings. Pirimiphos-methyl, in particular, demonstrated strong performance in areas with moderate frequencies of *kdr* and *Ace-1* resistance mutations, highlighting its potential for indoor residual spraying (IRS) campaigns in contexts of multi-resistance [39, 43, 44]. Studies conducted in Côte d’Ivoire and Burkina Faso have confirmed that pirimiphos-methyl, when applied to cement or mud walls, maintains residual efficacy for over 6 months, making it a robust candidate for seasonal interventions [44–47]. Similarly, bendiocarb continues to produce high mortality rates in several localities, despite the emergence of resistance linked to the *Ace-1* mutation particularly, where its frequency remains below 20% [48, 49]. These findings emphasize the importance of diversifying the insecticide classes used in vector control programs and prioritizing compounds that remain effective under high selective pressure. A comprehensive understanding of resistance mechanisms and their spatial heterogeneity is ultimately critical for optimizing interventions and sustaining the long-term effectiveness of vector control strategies in the face of escalating resistance. As recommended by the WHO, plant-derived molecules may also serve as an alternative tool against resistant mosquito populations [50–53].

*Anopheles arabiensis* plays a pivotal role in terms of contribution in malaria transmission due to its behavior and ecological distribution. Although it is less abundant, *An. coluzzii* exhibits a relatively high infection rate. This level of infectivity suggests an amplified local vectorial capacity, likely driven by strong anthropophily and favorable ecological conditions for parasite survival [54]. Recent studies have shown that *An. coluzzii* is particularly well-adapted to urban and peri-urban environments, with a preference for permanent and polluted larval habitats conditions that may sustain transmission even at low vector densities [55, 56]. In contrast, *An. gambiae s.s.* appears to play a minor role in this study, exhibiting a moderate sporozoite infection rate (SIR) and making a limited contribution to the entomological inoculation rate (EIR). This decline may be attributed to ecological replacement dynamics, as observed in the Kou Valley, where *An. arabiensis* becomes more prevalent at the onset of the dry season. It may also reflect increased selective pressure from the widespread use of insecticide-treated nets and indoor spraying, which differentially affect species within the complex depending on their feeding behavior and chemical susceptibility [57]. The spatial heterogeneity of EIRs observed across neighborhoods highlights the importance of local factors in transmission dynamics. Sabaribougou, which shows high infection rates across all three species, may reflect a combination of high vector density, limited control intervention coverage, and environmental conditions conducive to larval development. Conversely, Farakan and Dogona exhibit lower EIRs despite the presence of infected mosquitoes. This could be due to reduced vector density or better human protection particularly through the use of nets [45]. These findings call for a differentiated approach to vector control, one that accounts for species diversity, local ecology, and vectorial capacity. Targeted entomological surveillance and the adaptation of control strategies to the spatial and behavioral specificities of vector populations are essential to enhance intervention effectiveness.

## Conclusion

This study highlights a complex entomological situation in the city of Bobo-Dioulasso, characterized by high levels of pyrethroid resistance among malaria vectors and heterogeneous transmission dynamics across neighborhoods and vector species. The predominance of *Anopheles arabiensis*, its ecological and behavioral resilience, and the significant *An. coluzzii* infection rate at certain sites underscore the necessity of a localized and differentiated approach to vector control. The results confirm the strategic value of PBO-based synergized formulations, which demonstrate enhanced efficacy in contexts of metabolic resistance. Moreover, the continued susceptibility of vectors to organophosphates and carbamates particularly pirimiphos-methyl and bendiocarb provides robust operational alternatives for indoor residual spraying campaigns. In light of rising multi-resistance and persistent transmission in certain areas, it is crucial to strengthen entomological and molecular surveillance, tailor control tools to local resistance profiles, and establish integrated strategies to maintain the effectiveness of interventions. An understanding of the nuances vector ecology and behavior, combined with rational insecticide management, is essential to contain residual malaria transmission and steer public health policies toward sustainable solutions. One limitation of this study lies in the absence of multi-concentration testing. Additional assays using pyrethroids at concentrations 5 × and 10 × higher than the diagnostic dose are planned to further assess resistance levels and better characterize the tolerance profiles of vector populations.

## Data Availability

No datasets were generated or analysed during the current study.

## Abbreviations

*An*.: *Anopheles*
bp: Base pair
CTAB: Cetyltrimethylamonium bromide
DDT: Dichlorodiphényltrichloroéthane
DNA: Desoxyribonucleic acid
EIR: Entomological inoculation rate
F: Phenylalanin
HLC: Human landing catch
IRS: Indoor residual spraying
IRSS: Institut de Recherche en Sciences de la Santé
ITN: Insecticide treatment net
Kdr: Knockdown resistance
L: Leucine
LLINs: Long-lasting insecticidal nets
PBO: Piperonyl butoxide
PCR: Polymerase chain reaction
RR: Homozygous resistant
RS: Heterozygous
S: Serine
SIR: Sporozoite infection rate
s.l.: Sensu lato
s.s.: Sensu stricto
SS: Homozygous susceptible
WHO: World Health Organization
